# Rasburicase Plus Methotrexate Combination Therapy in a Case of Tophaceous, Difficult-to-Treat Gout

**DOI:** 10.7759/cureus.76265

**Published:** 2024-12-23

**Authors:** Maria Fernanda Pino-Zambrano, Patricio Cardoso Peñafiel, Enrique Calvo-Aranda

**Affiliations:** 1 Rheumatology, Hospital Universitario Infanta Leonor, Madrid, ESP; 2 Department of Medicine, Universidad Complutense de Madrid, Madrid, ESP

**Keywords:** case report, difficult-to-treat gout (d2t gout), methotrexate, rasburicase, tophi

## Abstract

Gout is a disease caused by the deposit of monosodium urate (MSU) crystals that produce joint inflammation and subcutaneous nodules (tophi). The treatment of gout aims to reduce serum uric acid (sUA) levels by administering urate-lowering therapies (ULT) such as xanthine oxidase inhibitors (XOI: allopurinol, febuxostat) or uricosurics (e.g., benzbromarone). However, some patients with tophaceous, difficult-to-treat (D2T) gout may not respond to or have poor tolerance of or contraindications to conventional treatment. Uricases such as pegloticase (PEG; polyethylene glycol-conjugated mammalian recombinant uricase), which degrades UA to allantoin (more soluble in urine), have emerged as a therapeutic alternative for such patients. The concomitant use of methotrexate (MTX) improves the efficacy and tolerance of intravenous PEG by reducing immunogenicity secondary to the formation of anti-PEG antibodies. However, this uricase is not marketed in Europe. Rasburicase, which is indicated for the treatment and prophylaxis of acute hyperuricemia resulting from tumor lysis syndrome in patients undergoing chemotherapy for hematologic malignancies, has been used off-label with some success in tophaceous gout. However, as with PEG, it is occasionally associated with potentially serious infusion reactions. We present a preliminary report on the use of Rasburicase plus MTX combination therapy in treating a single case of refractory tophaceous gout, highlighting potential areas for further investigation.

## Introduction

Gout is an autoinflammatory disease caused by the deposit of monosodium urate (MSU) crystals; the main risk factor is hyperuricemia, which is characterized by serum uric acid (sUA) levels higher than 6.8 mg/dl. Without proper treatment, it can lead to chronic joint damage and subcutaneous nodules (tophi). To dissolve these deposits, sUA values lower than 6 mg/dl (<5 in tophaceous gout) should be targeted [1]. As a standard of care (SoC), urate-lowering therapies (ULT) such as allopurinol, febuxostat, or benzbromarone are usually needed. Nevertheless, some patients with difficult-to-treat (D2T) gout (≥3 attacks/year and contraindication/refractoriness/intolerance to SoC) fail to respond to available treatments (consistent failure to reach sUA target, insufficient resolution of tophi) or experience adverse events with them, representing a therapeutic challenge and requiring different solutions [2]. Exogenous uricases replicate the function of the physiological uricases (catalyze the oxidation of UA into allantoin, which is more soluble in urine) present in other species, and they may represent a debulking therapeutic alternative for these patients [1,3].

Rasburicase is a recombinant Aspergillus uricase indicated for the treatment and prophylaxis of acute hyperuricemia in patients undergoing chemotherapy for hematological malignancies with a risk of tumor lysis syndrome [4]. It has also been successfully used off-label in patients with tophaceous and D2T gout [5-11]. However, it is associated with potentially serious infusion reactions due to immunogenicity, with frequent and rapid formation of anti-drug antibodies [10]. Pegloticase (PEG), another intravenous uricase, gained U.S. Food and Drug Administration (FDA) approval for use in uncontrolled gout. However, it can also lead to infusion reactions due to the formation of anti-PEG antibodies. This immunogenicity can also reduce the urate-lowering effectiveness of PEG [3]. In fact, the European Medicines Agency withdrew PEG's marketing authorization, and it is no longer available in the European Union [12,13]. Nonetheless, several studies have shown that the concomitant use of immunomodulatory drugs may improve the safety and efficacy of PEG when administered together. Indeed, combination therapy with methotrexate (MTX) has recently been approved for use by the FDA based on the MIRROR randomized trial [14]. To date, the use of this strategy has not been reported in gout patients treated with off-label rasburicase.

## Case presentation

**Figure 1:**
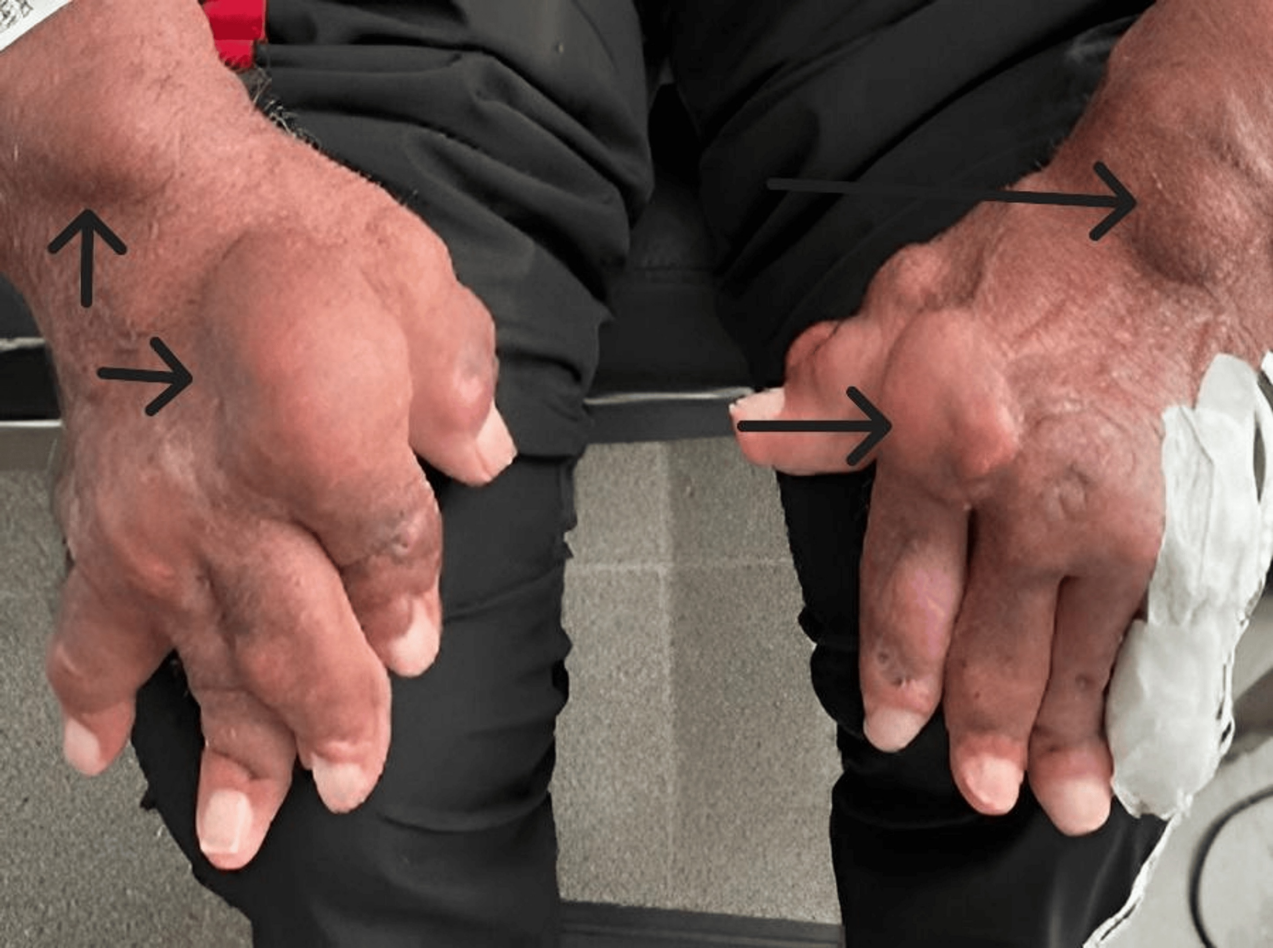
Patient with tophaceous D2T gout. Large MSU deposits (tophi) and multiple deformities in both hands. Black arrows indicate several tophi that were monitored during the treatment. D2T: difficult-to-treat; MSU: monosodium urate.

We present a 67-year-old male with early-onset polyarticular tophaceous gout first diagnosed when he was 20. His medical history included hypertension, type 2 diabetes, chronic ischemic heart disease (with coronary revascularization), and chronic kidney disease stage 3. Physical examination revealed multiple tophi on the upper and lower limbs (Figure 1).

Laboratory tests showed high sUA levels (10.8 mg/dl), elevated C-reactive protein (12 mg/L), and renal impairment (creatinine clearance 51 ml/min).

The patient had received several treatments, including colchicine, NSAIDs, and glucocorticoids, in addition to up to four different ULTs. He presented with refractoriness and poor tolerance, experiencing a rash with allopurinol, experiencing dizziness and vertiginous symptoms with both benzbromarone and lesinurad, and experiencing general malaise with febuxostat, even after multiple attempts at very low doses. He suffered from chronic pain and significant joint limitation. These conditions had a relevant influence on his quality of life and his perception of his body image.

Following a review of the literature and based on studies with patients treated with PEG in combination therapy with immunosuppressants, we proposed off-label treatment with rasburicase and MTX, since up to 40% of patients treated with uricase may develop anti-drug antibodies, with a loss of the sUA-lowering effect and an increased risk of infusion reactions [3,12,14]. Despite differences in half-life and immunogenicity between rasburicase and PEG, adding MTX to recombinant uricases in gout is a reasonable strategy, so in patients with uncontrolled gout receiving PEG (MIRROR), MTX increased the responder rate (sUA <6 mg/dL) at 6 and 12 months and reduced anti-drug antibody formation, as shown by pharmacokinetics data [14]. We received local authorization, and the patient was provided informed consent. Rasburicase was administered in 30-minute monthly intravenous infusions (0.2 mg/kg). The patient began subcutaneous methotrexate (10 mg/week, dose adjusted based on renal function; s.c. to facilitate adherence due to the need for multiple daily oral medications) and oral folic acid (5 mg/week) four weeks prior to the first infusion and continued during rasburicase treatment. Methotrexate was well tolerated throughout the follow-up period (total 15 months), with no increase in the number of infections or other adverse events (AEs). In addition, pre-medications (antihistamine, methylprednisolone) were administered before each infusion. The patient was closely monitored during and after each infusion. Blood tests were performed before each infusion and 24 hours later, controlling for sUA values and other parameters such as blood count, liver enzymes, or renal function. The size changes of the tophi were measured using a caliper.

**Figure 2:**
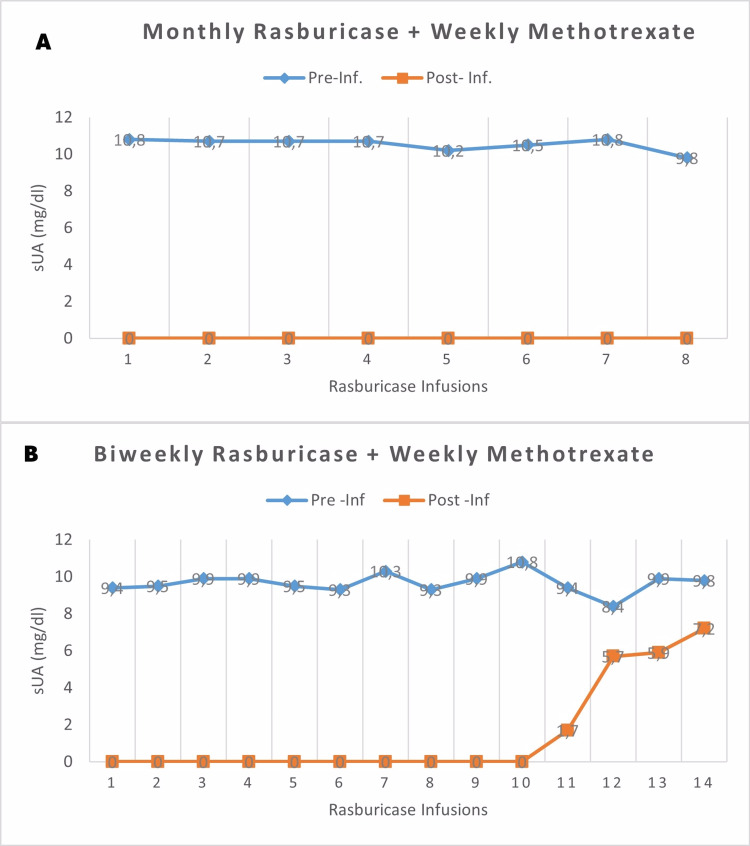
Serum uric acid levels over time in a patient with tophaceous D2T gout treated with rasburicase and methotrexate. Both panels show pre-infusion and 24-hour post-infusion serum uric acid levels during the two phases of rasburicase treatment: initially monthly (panel A), then intensified to biweekly (panel B) in an attempt to achieve a greater and more rapid reduction in the volume of the deposits. Pre-inf: pre-infusion (upper line); Post-inf: post-infusion (lower line); sUA: serum uric acid; D2T: difficult-to-treat.

The sUA levels decreased to an undetectable range 24 hours after each infusion but remained off-target before each new dose of rasburicase. Moreover, the tophi did not change significantly. Therefore, in the absence of gout attacks, infusion reactions, or other significant AEs, after the first six monthly treatments, the decision was made to intensify the treatment, switching to biweekly infusions. During the following months, the patient continued to tolerate the treatment well, with progressive improvements in joint mobility and the softening and partial size reduction of tophi (Table 1), some of which drained material spontaneously or during nursing treatments. However, pre-infusion sUA levels remained high after six months of treatment intensification, and a slight elevation of post-infusion sUA was evident, rising even higher over the next three administrations (Figure 2).

**Table 1:** Changes in tophi size during the first six months of treatment with combined Rasburicase and methotrexate therapy. The table illustrates the gradual reduction in tophi size (length × width, centimeters) at various anatomical locations in the patient during the initial months of treatment with rasburicase and methotrexate. 2MCP: second metacarpophalangeal joint.

Location	September 26, 2022	December 20, 2022	March 14, 2023
Right elbow	6 x 5.3 cm^2^	4.2 x 4 cm^2^	3.7 x 3 cm^2^
Left elbow	5.5 x 5 cm^2^	5 x 4.7 cm^2^	4.8 x 4 cm^2^
Right wrist	4.5 x 3 cm^2^	3.7 x 2.5 cm^2^	3.4 x 2.3 cm^2^
Left wrist	4.1 x 3 cm^2^	4 x 2.8 cm^2^	3.9 x 2.5 cm^2^
Right 2MCP	5 x 4.5 cm^2^	4.8 x 4.4 cm^2^	4.5 x 4 cm^2^
Left 2MCP	3.8 x 3.3 cm^2^	3.4 x 3.2 cm^2^	3 x 2.9 cm^2^
Right foot	3.3 x 3.2 cm^2^	3.2 x 3 cm^2^	2.9 x 3 cm^2^

Therefore, after 22 infusions, the treatment was suspended due to insufficient response and the risk of anaphylaxis.

## Discussion

Tophaceous D2T gout remains a significant therapeutic challenge. In patients with uncontrolled gout due to refractoriness/intolerance to SoC, uricases such as PEG or off-label rasburicase may offer a therapeutic alternative. However, potentially dangerous AEs have been associated with these drugs, mostly in the form of infusion reactions due to immunogenicity phenomena. The combination of immunomodulators, specifically MTX, with PEG, has been shown to significantly reduce immunogenicity and thus improve treatment efficacy and safety in gout. Although the half-life and immunogenicity profile of rasburicase is different from PEG (pegylated molecule with reduced immunogenicity, enhanced solubility, and increased serum half-life), PEG is not currently marketed in the European Union, and there have been no clinical trials that have assessed the use of Rasburicase plus MTX in combination therapy in patients with gout.

Our patient presented good tolerance to treatment during the first 12 months, with suboptimal efficacy and possible late-onset immunogenicity, given the occurrence of several consecutive measurements of pre-infusion sUA levels >6 mg/dL (a surrogate marker for the presence of anti-drug antibodies; Figure 2) [12]. On one hand, this could have been due to the shortening of the interval between rasburicase infusions, which was implemented after the first months with a monthly regimen (intended to reduce the large MSU deposits), or simply to prolonged drug exposure, as research indicates that antibodies against rasburicase were detectable in some patients after extended use [3,12]. Although we initially used a therapeutic regimen with monthly rasburicase infusions during the first months of treatment, in line with the exploratory study by Richette and Bardin (showing greater efficacy compared to a daily administration group), various reports have described diverse efficacy and safety outcomes with different intervals and doses of rasburicase in patients with gout [5-11]. Additionally, despite a theoretically greater bioavailability with subcutaneous administration and proper adherence confirmed by the electronic dispensing system, the dose of MTX may have been insufficient for this patient, lower than those used with PEG in the MIRROR trial (15 mg/week). However, the use of lower doses of MTX has also been reported in patients with CKD co-treated with PEG [15]. Lastly, it should be noted that although Rasburicase has a powerful hypouricemic effect, its serum half-life is less than 24 hours, limiting its impact on sustained sUA control [4]. This has led some authors to associate simultaneous treatment with other ULTs [7]. Unfortunately, this was not possible in our case due to multiple intolerances. Remarkably, our patient did not suffer gout attacks during the combined treatment, unlike all the other reports of patients treated without MTX [5,6,8-11].

## Conclusions

Tophaceous D2T gout is not uncommon and remains a therapeutic challenge. In patients with uncontrolled gout, uricases may offer a therapeutic alternative, but their efficacy and safety may be affected by immunogenicity. This is the first case of non-oncological use of rasburicase plus MTX combination therapy in a patient with tophaceous D2T gouty arthritis. While initial tolerance and partial efficacy were observed, the therapeutic effects were not sustained, ultimately leading to treatment discontinuation. The potential for immunogenicity with Rasburicase, as seen in the declining response in this case, may limit its long-term use, aligning with the trends observed in uricase therapies. So, this treatment may be considered in specific, refractory cases, but with close monitoring. Future research should investigate optimal dosing, administration intervals, and the role of immunomodulation in enhancing the long-term efficacy of rasburicase or similar uricase-based therapies.
